# Editorial

**Published:** 1855-06

**Authors:** 


### THE MEDICAL EXAMINER.

### PHILADELPHIA, JUNE, 1855.

### AMERICAN MEDICAL ASSOCIATION.

Our readers will find an account of the proceedings of the last session of the American Medical Association in the present number. It will be seen that we have omitted the names of all the special committees from whom no reports were received, as well as the names of those appointed to report at the next session. We have also been unable to give the eloquent address of Mayor Conrad at Independence Hall. We regret the latter circumstance the less, however, as, besides a resolution passed by the Association to publish the address in the next volume of the Transactions, it has been very largely distributed to the profession in pamphlet form, by means of the Committee of Arrangements. With these exceptions, we believe that our account of the proceedings will be found to be a very full and accurate one.

Unanimity and good feeling prevailed throughout the session.—Not a single contre-temps occurred to mar the general harmony of the proceedings—all things went smoothly as a marriage bell. The only subject to be regretted was the rather singular circumstance that the three most important committees of the Association—the Committee on Plans of Organization for State and County Socie. ties, the Committee on Medical Literature, and the Committee on Medical Education, all of which were anxiously looked for—gave in no reports. We sincerely trust that we may never hereafter have to record a similar omission.

We cannot refrain from copying the following very courteous and flattering remarks from the graceful and ready pen (we presume) of Dr. Morland, one of the editors of the Boston Medical and Surgical Journal. We feel very sure that our readers will be much gratified by a perusal of them.

*The American Medical Association.*—The session of this body, which has just closed, while it was characterized by the most unbroken harmony in all its proceedings, and by the great value and importance of the scientific communications made, was also, we will venture to affirm, unsurpassed by any yet holden, in the number and 'variety of the objects of interest exhibited to the members of the Association, and in the generous hospitality shown them during the whole of their stay in the beautiful city of Philadelphia.

We have already alluded, in a somewhat desultory manner, to the business proceedings of the session—and gratefully remembering the numerous attentions shown, and the hearty welcome everywhere extended, we wish to put a few recollections upon record. To the "Committee of Arrangement" the thanks of the Association are eminently due, for the complete and efficient discharge of their multiform and onerous duties. So quietly and smoothly did everything move on, that while we were well aware how much time and attention this management must have required, we daily admired the tact and good judgment which so unostentatiously, yet so thoroughly, directed the whole. Dr. Hays, the Chairman of the Committee, was certainly never *more* successful in any of his undertakings, and this, as every one will allow, while it is saying a great deal, expresses not one whit too much. We would particularly notice the great utility of the handsomely-prepared volume, a copy of which was presented to each member of the Association who duly registered his name. This little book contains the names of the Officers of the Association; of the Committees who were to report; the "code of ethics" of the Association; short accounts of places of interest to be visited; a map of Philadelphia, &c. &c. A publication of this sort is almost indispensable at such a time.

The delegations from the several States (twenty-six States being represented), were very full; five hundred and twenty-three, we believe, is the entire number of registered names. The members who so inclined were conveyed in commodious coaches, sixteen in number, to visit Girard College and Fairmount Water Works. At the College they were kindly received by President Allen, who conducted them over the magnificent and completely appointed building—the boys, in two or three of the school-rooms, being kept sitting for a while, that the visitors might pass through and inspect them. Every one must have been impressed by their neat, orderly and contented appearance. Great credit is due to the managers of this Institution and to its officers generally. Not the least pleasant part of this visit was the ascent of the members, *en masse*, to the roof of the building, whence a most imposing and extensive view of the city and its environs is obtained. The solidity of the building, and its faithful and costly construction, are worthy of special note. It is well known that the *roof*, even, is of stone, and of immense strength and weight. We remarked but one individual who hesitated to ascend, and he, indeed, retired, seemingly apprehensive that the additional weight of the visitors might be too much for the supports of the roof! We are happy to state that the latter did *not* fall in! The medical wisdom of the land escaped entombment beneath *that* marble!

Several other excursions were made, with universal satisfaction;—to the Philadelphia Hospital, an immense establishment, comprising within its walls a lunatic hospital, admirably managed; paupers are the chief inmates of this institution; the area covered by it, is, we were told by the gentlemanly and efficient resident physician, Dr. Campbell, *sixteen acres*. The amount of labor done by Dr. C. and his assistants must be very large, and, so far as we could observe, nothing was neglected.

The reception of the Association in Independence Hall was exceedingly gratifying. To visit this famous spot, in itself alone, is no slight privilege; to be eloquently greeted, and made to feel "*at home*" in it, is much more. We are happy to know that the beautiful Address of his Honor, Mayor Conrad, is already printed, in conjunction with the appropriate presentation speech of Dr. Hays, and that they both will be incorporated in the next volume of "Transactions."

We refer with great pleasure to the various elegant and delightful entertainments offered to the Association, by several of the physicians of Philadelphia, some of whose houses were thrown open every evening, and a hearty welcome given to all the invited guests. When the numbers of the Association are remembered, the extent of this noble hospitality may be, in some degree, estimated. Not only was the outer man most sumptuously provided for, but the lovers of the fine arts and of scientific rarities were gratified to the full. The very valuable conservatory of Dr. George B. Wood, President of the Association, was lighted for the inspection of visitors, with the gardener in attendance. This rare collection is chiefly composed of medicinal plants, and was gathered, as we learn, by its owner, for the purpose of illustrating his lectures. Drs. Wood, Bache, Hodge, Jackson, Pancoast, Norris, Paul, Alfred Stillé and Hartshorne, sent cards of invitation to the members of the Association, who gladly accepted, and seemed to have attained the *ne plus ultra* of enjoyment at each of their entertainers' houses. For ourselves, we are heartily glad that no public entertainment was given to the assembled guests, for, aside from the enormous and unjustifiable expense incurred, and which, of itself, would finally defeat the objects, and virtually annul the meetings, of the Association, it would be disgraceful to have a renewal of certain scenes that have transpired. We hope, therefore, that the example of our Philadelphia brethren will be followed hereafter. A more generous, elegant and *appropriate* hospitality we have never witnessed.

The Association may consider itself highly complimented by the cordial reception given them by Mr. Lea, of Philadelphia. Having an ample fortune at his command, he has indulged an exquisite taste, by the accumulation of a large number of exceedingly valuable paintings and other works of art, and these are admirably arranged in his really magnificent mansion. We can say with truth, that it is worth a journey to Philadelphia merely to see these treasures. When, in addition, all that a courteous gentleman could devise, to please his visitors, is done, we cannot too gratefully express our acknowledgments. Mr. Lea was assisted in doing the honors of his house, by his son (of the well-known firm of Blanchard & Lea), to whom our thanks are especially due, for very pleasant attentions. Upon so agreeable a theme, we might fill a far larger space than we can command. Many gentlemen (ourselves among the number), can testify to the polite invitations to private tables and to the delightful intercourse of family circles. Under such *treatment* we prognosticate a decided proclivity on the part of all present, to fall into the same way again! And we make no doubt that our friends of the far North-West, will, next year, welcome the Association in the warmest manner and with the happiest anticipations. We can testify to the very decided wish (we might almost term it *will*), that Detroit, Michigan should be the next place of meeting—and which was finally so settled. Nashville, Tenn., contested the matter bravely. This eagerness upon the point, clearly shows that the profession throughout the land feel the beneficial influence of the "American Medical Association."

During the latter half of the session, Dr. Storer, of this city, circulated among the members copies of the following stanzas, sent to him by Dr. O. W. Holmes, in expectation of a public entertainment being given. Having by no means been so generally seen as their beauty and appropriateness deserve, we give them, in this pleasant connection, by permission of their author:

A TRIPLE health to Friendship, Science, Art,
From heads and hands that own a common heart!
Each in its turn the others' willing slave;
Each in its season strong to heal and save.

Friendship's blind service, in the hour of need,
Wipes the pale face—and lets the victim bleed.
Science must stop to reason and explain;
ART claps his finger on the streaming vein.

But Art's brief memory fails the hand at last;
Then SCIENCE lifts the flambeau of the past.
When both their equal impotence deplore—
When Learning sighs, and Skill can do no more,
The tear of FRIENDSHIP pours its heavenly balm,
And soothes the pang no anodyne may calm!

*May* 1, 1855.

And thus, under the most favorable auspices, without and within, closed this, the eighth annual meeting—nothing wanting, not even the touch of poesy, to complete and preserve its agreeable reminiscences.

---

The readers of the Examiner may remember that we published in the April number a review of Dr. Eben. Watson's work "On the Topical Medication of the Larynx," &c., in connection with a paper by Horace Green, M.D., "On the Employment of Injections into the Bronchial Tubes and into Tubercular Cavities of the Lungs." A few days after the issue of the journal, we received a note from Dr. Green, asking us, on the ground of injustice to himself and to medical science, to allow him room in the next number to correct in a brief article certain mis-statements made in the review. We answered him immediately that he was at liberty to correct any mis-statements of facts that might have been made, and in the manner he proposed, viz., in a short article. It may be proper here to observe, that this always has been our rule. We claim, however, to be the judge whether the allegations are mis-statements or are not, and for the proper exercise of such judgment hold ourselves responsible. On the other hand, when an article is signed, we do not consider ourselves accountable for anything either said or implied in it. To its author alone belong the right and responsibility of correcting any mis-statements in it, and that right no editor can properly refuse him. On the 14th of April we received a lengthy paper from Dr. Green, with a note accompanying it, thanking us for our compliance with his request. After a careful perusal of the paper, we returned it to Dr. Green, stating our reasons for so doing in the letter which will be found below. A large portion of the paper was entirely irrelevant. The introduction into it of Dr. Ware's favorable opinions of Dr. Green's practice, in connection with a review written years ago in the American Journal of Medical Sciences, and the account of the unde derivatur of Dr. Watson's topical treatment of hooping cough and other diseases, though, no doubt, exceedingly interesting, were matters which had nothing whatever to do with the correction of mis-statements asked of us. With every disposition, therefore, to make allowance for Dr. Green's desire to vindicate himself from what he considered misrepresentations, we thought the reasons just mentioned, with those given in our letter to him, were such as obliged us to decline his paper. To have published it, would have been a withdrawal of what we were satisfied was correct and not unjust. It afterwards appeared in the May number of the American Medical Monthly, a journal of which Dr. Green is a co-editor, under the heading of "The Examiner corrected." In an editorial in the same journal are the following remarks: "The other answer explains itself. It was offered to the Examiner, in which the review appeared, but was rejected, for reasons which appear to us quibbles. The editor or the reviewer has shown himself a little thin-skinned, as the saying is, in not being willing to allow his readers to hear the other side. However, that is none of our concern, and we merely mention it as a passing reflection. Meantime, we must do what we can to perform the duty which properly devolved upon the Examiner."

Our readers can judge for themselves, after reading the following, whether the term "quibble," as applied to our refusal, was either just courteous.

*Philadelphia, April 17th,* 1855.

HORACE GREEN, M.D.

*Sir,*—In answer to my letter to you of the 6th inst., in which it was stated that I would cheerfully allow you an opportunity of correcting, in the manner you desired, (in a brief article,) any mis-statements of facts made in a review contained in the April number of the Examiner, I received yesterday a note from you accompanying a manuscript of twelve pages. After carefully reading it over, and comparing it with the review and Dr. Watson's work, I have come to the conclusion to decline its publication. This I do, from having fully satisfied myself that, with one exception, the matters complained of are *not* mis-statements. The exception I refer to, which I shall take the earliest opportunity to correct, relates to the passage, (p. 205 of the Examiner) in which the reviewer speaks of a sponge "dropped into the windpipe of a man, whose larynx Dr. Peaslee was cauterizing." This mis-statement, which I believe, which I may say I know, from the character of the gentlemen who wrote the article in question, to have been unintentional, is the only one, which, after a most careful examination, I have been able to discover. As I, however, wish to show, from courtesy to you, on what grounds I make this statement, I will briefly go over the various points to which you have addressed your letter, premising that the Reviewer's statements and not his opinions, are the matters in issue between us.

The first mis-statement you complain of, is the remark that a "sponge, which, according to Dr. Green, dropped into the wind-pipe," &c. (p. 205 Examiner.) This assertion was an incorrect one, it is true. It is plain, however, from the context, that the Reviewer's object was not to condemn Dr. Peaslee for letting a sponge drop into the larynx—such an idea never entered into his mind—but was to show his readers what was the opinion of Dr. Watson of the size of the sponge extracted. Other journals and foreign ones have quoted, I may observe, this same passage.

On page three of your manuscript, you say, after giving an account of the same sponge case, "Now it is the above statement, &c., that your Reviewer, along with Dr. Watson, would characterise as being 'grossly inaccurate.'" It is a sufficient answer to this, to say that the Reviewer used no such language. He expresses no opinion of his own upon the subject. The only statement made is in Dr. Watson's own words, (p. 20, English edition.) In the original from which it is taken, it is followed by remarks, I perceive, far more severe than any copied into the review. Dr. Watson says, "when once the mind of the reader has been impressed with the gross inaccuracy of observation, or the still more inexcusable laxity of description which characterizes the statements above quoted, it is in vain to attempt pointing out the grain of wheat in the bushel of chaff, &c." I cannot conceive that the Reviewer is bound to defend these opinions of Dr. Watson. It should suffice, that he gave what he did copy, truly.

As regards your strictures upon the statement of the Reviewer, that Dr. Watson is "altogether opposed to its employment, (speaking of the use of caustic in croup,) unless before any exudation has been poured out, which is equivalent to saying, before there is any croup to treat," you, yourself, admit that Dr. Watson *does* object to its use during the stage of exudation. (See p. 53, Watson.) He does not do this, however, for theoretical reasons alone, as you state, but from positive experience in its use. He narrates two cases, and remarks (p. 46) that he "could relate several others, the subjects of which were children," to prove that the topical treatment is unsuitable during the acute stage, and that it retards, if it does not prevent its favorable progress. As to the treatment of the disease *after* the exudation is cast off, Dr. W. does, as you say, consider "the renewal of the topical treatment as one of the appropriate measures to be employed," "but it is only after *blistering*, *gentle purgatives*, with *tonics*, a *nourishing diet*, and as soon as the effects of the *calomel* permit," that he mentions it.

In my opinion, the Reviewer had a perfect right to use the words, "which is equivalent to saying before there is any croup to treat." Every one is satisfied, when the membrane has been seen, that a pre-exudative stage must have existed. It is a very different matter, though, to determine from symptoms, that such will be the termination of the disease. We may suspect that such will be the issue, and may imagine, if we please, that our treatment has been successful in preventing it. When we see, and only when we see, the membrane, can we be sure, at least such is my opinion, that we are treating a case of that terrible affection.

All that portion of your manuscript which relates to Dr. Ware and his opinions, is entirely irrelevant to the matter at issue.

As regards the Reviewer's remark, when upon "follicular disease of the larynx," that Dr. Watson is disposed to regard it altogether as what Carlyle would call "a sham." I must refer you, for its defence, to pages 66 and 67 of Dr. Watson's work. I shall make but one extract. The author remarks, "The latter (follicular disease) must, indeed, be an affection of very rare occurrence, since so few writers take notice of it, and I feel almost inclined to question the reality of its existence as a disease of the larynx in any case. At all events, I do not hesitate to assert, that we have no evidence, in even Dr. Green's writings, which is worthy to be considered as establishing the matter; and I will also maintain that the assumption of the existence of such a lesion as the cause of certain symptoms, is neither necessary nor philosophical." This statement clearly shows that Dr. Watson not only doubts, as you allow he does, the *extent* of the disease, but also "the reality of its existence."

Finally, as respects the remarks of the Reviewer upon "cauterization in hooping-cough," as he no where denies that Dr. Watson owes his first trial of it, in that affection, to a perusal of your work on "Diseases of the Air Passages," you, surely, have no right to blame him for what he has left unsaid.

These are all the points I believe, in issue between us. Your manuscript, however, is so largely taken up with other subjects, entirely irrelevant to the question, that I should be obliged to decline it for such reason alone, were there none others.

In justification of the necessity of a review, I would state that your position as an author, a professor, a physician in large practice, and a co-editor of an extensively read journal, demanded a thorough examination of the novel procedure brought by you before the profession in your article "On the Employment of Injections into the Bronchial tubes and into tubercular Cavities of the Lungs." I was in hopes, after reading it, that it would be properly noticed in some of our journals. As it was not done, however, and as I conscientiously thought that your advised proceeding must be both hazardous at the time it is employed, and liable to be followed by dangerous consequences, I requested a review of it, and can only express my sincere regret that the opinions therein expressed were not more acceptable to you, since no personal feelings were in any way engaged in the discussion. I remain, very respectfully,

S. L. HOLLINGSWORTH.

---

We copy the following remarks from the Charleston Medical Journal, as the person to whom they refer is now in our city.

DR. ALEX. TURNBULL.—In answer to numerous inquiries which have been made concerning the scientific attainments of Dr. Turnbull; and, also, to correct the erroneous impressions which have gone abroad through the medium of the public prints, we undertake an exposition of his career; in doing which, we are deviating from our accustomed course, and giving undeserved importance to one who would not otherwise have occupied our attention. The unblushing assumptions of this person to perform impossibilities, and the rumours of his pretended cures, have so impressed the minds of the credulous, that it becomes our duty to warn our readers, who may have friends or patients laboring under diseases of the eye or ear, from crediting the recommendatory advertisements which have been in circulation, respecting his "simple and painless processes," by which diseases hitherto beyond the resources of art are speedily and permanently cured

Many an anxious parent has been subjected, on the faith of these advertisements, to serious inconvenience and considerable expense, in the anticipation that soon their deaf-mute children would be restored to the healthful use of their dormant faculties. From day to day they were encouraged to hope, until, wearied by a longer attendance, or being assured that time alone was required to perfect a cure, they have departed in trembling anxiety, too soon to have the unwelcome truth forced upon them, that the objects of their solicitude were as hopelessly incurable as ever.

Some time since, Dr. Turnbull came to this country, and soon rumours reached us of his curing all manner of *deafness and blindness;* in due time Dr. T. reached our city, and took rooms at one of our hotels, where he announced himself as being prepared to treat the diseases of the eye and ear. For a time, he attracted very little attention; but, after treating divers cases, two were announced in a respectable city paper, (Charleston Courier,) as having been cured by his peculiar processes.

"One, a boy from Georgia, now nine years old, who had lost his hearing from fever in his third year," and been since that time completely deaf, "has been restored to an unusual degree of delicacy and sensitiveness, and he is making incredibly rapid progress in distinct articulation." Now we know this to be the reverse of the fact. Both parents of this child have emphatically averred that his hearing is not in the slightest degree restored; and, on seeing the above advertisement, the father became indignant, and immediately telegraphed the Augusta papers not to copy it.

"The other case is a youth from this city, aged 18, whose infirmities (of hearing and speech) being in some sort hereditary, and of a most decided character, afforded a crucial test of Dr. Turnbull's processes. In this interesting case, also, the progress of improvement has been rapid and uninterrupted, and the patient is now hopefully undertaking the education which is necessary to give him the due use and appreciation of his new born senses."

This case is the only one, that we know, which has been benefited. But there is no miracle here. There was only an absence of secretion in the external meatus, and the sense of hearing was obtuse, and was rendered less so. The patient has been under treatment for several weeks, and is not yet discharged.

We are informed, in the same Journal, that Dr. T. has contributed *largely* to the leading Medical Journals of England, and that "his professional status has been recognized and approved by the most compretent European authority, and the value of his discoveries and contributions, in medical resources and remedial agencies, has been cheerfully admitted and abundantly attested."

Before commenting on these statements we will sketch the history of the Doctor, and present the opinion of the profession in England concerning him.

In 1835, there appeared, in London, a duodecimo of 171 pages, "*On the Medical Properties of the Natural Order Ranunculaceæ and their Alkaloids, Veratria, Aconitine, Delphinia and Sabadilline.*" By A. Turnbull, M.D.

The reviewer of this work, in the "British and Foreign Medical Review," says that "if one quarter of what it contains be true, (and surely that would not be an unreasonable requisition,) Dr. Turnbull's alkaloids are sufficient to change the face, not only of medicine, but of society."*

"Of nine cases of affections of the heart, veratria is said to have cured

*The latter remark having reference to the moral effects which would ensue from the prolongation of human life, and the eradication of a large class of incurable diseases.

or relieved all; of thirteen cases of neuralgia, to have cured twelve; of nine cases of rheumatism, to have cured eight, and almost the remaining one. The heart affections, moreover, were evidently cases of organic disease, though the author, with great humility, generally refrains from saying so. * * * * Every case of neuralgia was perfectly cured, except one, and even of that the history concludes, in the text, with the words 'leaving no trace of the affection behind, neither has any renewal of it taken place?' (p. 70.) The note, however, a traitor to the text, says—'In this instance the veratria has completely failed in giving permanent relief,' &c." (*B. and F. Med. Review, vol. ii, p.* 499.)

These alkaloids were given a fair trial by the profession of that day, but no such effects followed their use as had been attributed to them by Dr. Turnbull. Veratria and Aconitine have been extensively employed down to the present time, and, though they sometimes are found useful as topical applications in neuralgia, like other medicines of their class, they are, internally, still of doubtful efficacy.

*A "Treatise on Painful and Nervous Affections, and on a New Mode of Treatment for Diseases of the Eye and Ear,"* by A. TURNBULL, M.D., appeared in 1837. The treatment of the affections of the latter organ, comprised in a few pages, consisted in the application of the alkaloid veratria to the external meatus and the adjoining parts. The "electro-stimulation," as the author styles it, having proved so efficacious in curing deafness, he determined to experiment upon the deaf and dumb, and soon cures were announced as having been effected by this means. The possessor of this remedy betook himself to Scotland to operate upon the deaf-mutes of that country, but he was not so successful as might have been expected; still rumour said that he had cured several cases in the Deaf and Dumb Institutions of Edinburgh, and it was not until some time had elapsed, that Mr. ROBERT KINNIBURGH, the intelligent head master of that institution, publicly asserted that no cure had been effected.* His cures were also questioned, and his statements severely criticised in Chambers' Journal, for September, 1839; but, three years afterwards, we find the Edinburgh Journalist retracting his former strictures, and lauding the achievements reputed to have been performed by Dr. T. in his tour through Scotland.

The editor tells us, that wishing to arrive at a thorough conviction, he "studied the most recent and approved works on aural surgery, in order to ascertain in what respects Dr. Turnbull's practice differed from that which was general in the profession." Now it is impossible to understand, or appreciate the treatment of a class of diseases, until the anatomy, physiology and pathology of the organs involved, have been thoroughly studied. An unprofessional man cannot be convinced of his error, or justly estimate the merits of a cure, or the causes of failure.

Here is what this observer relates:

"Experiment in time showed that in cases of deafness arising from low nervous energy, a class to which nearly all the cases of the deaf-

*Vid. Wilde's, on Diseases of the Ear; Am. Ed., p. 55.

dumb belong, the organ may be more or less successfully treated by applying to it a weak alkaloid. This he rubs gently on the tympanum by means of an instrument tipped with chamois leather, and generally in ten minutes the effects are manifest.

"But the greater number of diseases of the ear, arise from cold, producing acute and chronic inflammation, and diminishing or altogether obstructing the flow of wax, whereby the tympanum and other parts of the outer ear, from being exposed to the air, are thickened, and so deprived of sensibility. Cold also produces inflammation and consequent accumulation of mucus in the passage called the Eustachian tube, which communicates between the internal ear and the back of the mouth. Finding cured persons relapse in consequence of the defect of wax, Dr. Turnbull was prompted to use his ingenuity in endeavoring to discover the means of sustaining that secretion. He reflected that the application of the mouth of the child to its mother's breast, by removing the pressure of the atmosphere, causes the milk immediately to flow, and he conceived that a similar result might follow with respect to the wax of the ear, if he could by any means remove the pressure of the atmosphere from the external parts. For this purpose he at first used a syringe with an India-rubber mouth, exactly fitted to the aperture of the ear. The plan was successful; the blood vessels resumed a free circulation, and the flow of wax recommenced. It strikes us that we have rarely known a more beautiful instance of a simple natural principle being taken advantage of by human ingenuity for a human end."

Had this writer profited by his studies, he would have known that there is no analogy between the mammary gland and the follicles of the meatus, or between their respective secretions. The flow of milk from the former, by the removal of atmospheric pressure, is in obedience to a physical law, while the secretion of the latter depends upon the normality of the secreting follicles, and is poured out independent of any atmospheric condition.

Again :—"The clearing of the Eustachian tube, for which no means formerly existed but the application of medicines to the bowels, (!) or the *dangerous* use of the catheter, was effected by Dr. Turnbull by the same simple means."

Here we have a veracious, but misguided man, stating his convictions, and imparting to the world the value of remedial agents, about which he really knows nothing; showing too clearly, by his laudation of Dr. T., that he was the dupe of that person. Catheterism of the Eustachian tube is now well known to be a safe and simple operation. From the time of its first performance by Guyot, to the present day, it has been so considered.* We have seen it performed scores of times by the surgeons of Great Britain and the Continent, and have never known any unpleasant consequences ensue. But we need not be surprised that the friends of Dr. T. should call it a *dangerous* operation; for the only two cases recorded of injury having resulted from this simple procedure, occurred to Dr. Turnbull himself, *two of his patients having fallen victims*

*Vid. Pilcher on the Ear. 1843. p. 258.

*to it, and on both of whom coroner's inquests were held.* One of these, aged 68, was, almost immediately after the operation, attacked with a violent swelling in the throat, of which he died on the tenth day; the other, aged 18, in the enjoyment of perfect health, *died whilst undergoing the operation.* Messrs. Liston, Quain and Savage, were present at the post mortem examination, and stated, as their opinion, that death was caused by the forcible injection of cold air into the tympanum."*

After this sad affair, Dr. Turnbull resorted to a more "simple means" of clearing the Eustachian tubes, as indicated above. This he does by means of an air pump, in connexion with a flexible tube, "introduced into the mouth of the patient, and applied to the orifice of the Eustachian passage. A communication is thus opened between the previously rarified air in the receiver and the orifice, from which a discharge of mucus is made to enter the tube, which is then withdrawn."

In 1841–'43, Dr. Turnbull is again heard of in new fields of discovery. The Literary Gazette, whose glaring advertisements, on a former occasion, attracted the attention of the Edinburgh Journalist, is again in the van; and we read of *cures of blindness by the fumes of Prussic acid.*

We will let the writer speak for himself:

"The various stages of cure, advanced in our presence, by the simple application, for about half a minute, or until a little warmth was felt by the patient, of the vapor of hydrocyanic acid in a small phial, held up to the eye, with an aperture fitting the form of that organ; the various nature of the diseases so assailed—opacities of the cornea, inflammation, cataract, amaurosis, iritis, &c., &c.; the various stages of relief which the patients had reached, with sometimes one eye opened to sight and pleasurable to look upon, and the other left nearly blind and in its pristine deformity, to show what had been achieved; the various appearances of films removing, cataracts breaking up and being gradually reabsorbed, pupils being re-developed, and other altogether extraordinary symptoms of remedy and regeneration, filled us, we repeat, with wonder and delight."

The editors of Chambers' Journal considered the name of the editor a sufficient guarantee for the accuracy of these statements, and at once began to sound Dr. T.'s praise abroad. Their puffs and notices, spread over many pages of their Journal, may be viewed as a propular report on Ophthalmic and Aural Surgery.

Dr. T., emboldened by such commendations, published a short letter in the Lancet, (Sept. 28th, 1841,) containing a few "hints on the effects of hydrocyanic acid upon the Eye." His pamphlet, a small octavo of eighty-nine pages, on the "*Treatment of the Diseases of the Eye, by means of Prussic Acid Vapor, and other Medicinal Agents,*" appeared two years later. Dr. Turnbull having had a little practice in authorship, now indulges in theory, and, in his own (original) way, attempts an explanation of the *modus operandi* of the medicaments he prescribes.

*Lancet, July 6th, 1839; also, London Times same date.

"It is a well known fact," he says, "that the eyes of those who have been destroyed by hydrocyanic acid, for a length of time after death show none of the usual symptoms of dimness; on the contrary, the eye is clear and the pupil much dilated. This satisfied me that the acid exerted a specific action upon the eye, which might be made available, as a medical agent, for relieving many of the diseases to which that organ is so subject"—(p. 4.)* The condition of the eyes, after death, of those who have died from the effects of Prussic acid, are well known to be as Dr. T. describes, but the inference drawn from the fact is preposterous and absurd: for, as those who die from the effects of carbonic acid, or those who expire in a fit of apoplexy, present the same appearance of the eyes, we may, by the same analogical mode of reasoning, infer that the fumes of charcoal, and slight congestion of the brain, are specific remedies for diseases of these organs.

The explanation of the therapeutic process is as follows:—"The plan I generally adopt is to put into an ounce phial a drachm of the acid, and hold it in close contact with the eye, the eyelid being open, for the space of about half a minute, or until such time as the patient feels a little warmth, or the person holding the phial sees the pupil greatly dilated and the vessels of the eye injected with blood, which is the invariable effect of the application of the acid. The patient is not sensible of pain from this peculiar state being induced, which appears to me to result from the powerfully sedative influence of the acid, thereby showing two opposite powers, to wit: stimulating and sedative, are exerted at the same time, and thereby the uneasiness, arising generally from a stimulant alone, is prevented. Its great power in removing those diseases chiefly arises from the two powers being so blended, and thus enabling the eye to bear a sufficient stimulating action without injury. *The person who holds the acid to the eye should be careful not to allow the patient to smell it.*"†

This is original indeed! Pain is avoided, while incipient inflammation ("the vessels being greatly injected") is produced, which cures cataract, iritis and amaurosis. Diseases essentially different, resulting from diverse causes, and thus cured by stimulation, carried further than could otherwise be done by the sedation, which is also an effect of the vapor! But why, we may inquire, is the bottle-holder warned to remove the phial, for fear of too great an impression being produced, if the two forces are antagonistic and of equal power? In accordance with the above theory, it may be suggested that the forces will all necessarily counteract each other, and no effect will be produced.

Dr. T. uses the essential oil of bitter almonds in the same diseases, feeling convinced, he says, that it is more soothing, relieving pain without dilating the pupils or causing much redness of the eye, and being useful, also, in taking away the heat occasioned by the hydrocyanic acid. We may ask, if this harmless remedy fulfils all the required indications, why use the most dangerous? and why, on the same page, assign the

* Vid. Lancet, Oct. 9th, 1841.

† Vid. Lancet, as above.

same reasons for preferring and rejecting each alternately? But the Doctor is not satisfied yet; he has discovered *newer* remedies, and must consequently apologize for their introduction, which he does by disparaging his former favorite.

One reason why he did not rest satisfied with the great effects produced by the Prussic acid was, that its action, like that of all other medicines, decreased in power by continued application, thereby rendering it necessary to have occasional recourse to other medicines, in order to ensure a more *speedy recovery.* Another reason was, the reluctance of many individuals to submit the eye to the action of so potent a medicine. Well might a prudent person object to allow the vapor of concentrated Prussic acid to be brought in contact with his eyes, when such authorities as Pereira and Christison state, that, if a drop of the pure acid be placed on the throat of a dog, or applied to the eye, death takes place in a few seconds; and that, inhaling the vapor, decidely produces death more quickly than any other mode of using the acid.

The *newer remedies* are the *chlorocyanic and the sulphuretted chyazic acids,* the *chloruret of iodine,* and the *bisulphuret of carbon.* The first two articles are very different in their action from the Prussic acid, however, but we are to infer that they produce the same effects. The vapor of the *chloruret of iodine* irritates the eye, and the *bisulphuret of carbon* causes an intense prickling sensation, yet for reasons known only to Dr. T., they all cure "*blindness.*"*

The following is the ingenious explanation of the *modus operandi* of these substances when applied to the skin: "The action of these medicines, which contain so large a share of carbon, arises from the carbon in the vapor permeating the cuticle, and coming in contact with the oxygen in the vessels, which is conveyed through every part of the frame by inspiration and otherwise, thereby forming carbonic acid gas, which evolves heat in the ratio of the quantity consumed by the oxygen." On page 70 of his work last noticed, he says, "the rationale, in my opinion, is, that the large quantity of carbon in some of the essential oils, and its solubility in alcohol, permits it, by friction, to pass through the cuticle and unite with the oxygen."

The long received theory of the cure of amaurosis by stimulating the nerves of the skin, is then no longer tenable after the above lucid explanation.

We have seen that Dr. Turnbull's "simple means" for the cure of deafness, was sufficient to cause the death of two of his patients. But since he has resorted to the vapor of concentrated Prussic acid, as a local application to the delicate surface of the eye, we hear of nothing more serious than frequent failures, some of which necessitated the refunding of his fees.†

From our references, it has been seen in what repute Dr. Turnbull is held, and what has been considered the value of his medical contributions, by his own countrymen. We have not particularized the unpro-

* Vid. Lancet, Oct. 29, 1842.

† Lancet, Jan. 7, 1843.

fessional and disreputable acts of Dr. Turnbull, which we find recorded in the medical periodicals of his country; for so numerous have they been, that it would look like persecution to repeat them here; and our readers would have cause to complain of the wearisome length of the sketch we have drawn.

But before dismissing the subject, we must advert more particularly to some of the pretensions of Dr. Turnbull, and the results of his practice as it has come under our observation. He not only attempts to cure deaf-dumbness, but has endeavored to force conviction that such has actually been accomplished. Now, congenital deaf-dumbness has always been regarded as incurable, and we have no satisfactory proof that a single deaf-mute has ever been cured—that is to say, has so recovered the use of the organ of hearing as to be able, under all circumstances, to communicate with other persons in an unrestrained manner.*

But, when this condition has been produced by some mechanical impediment, or by some pathological state of the organ or derangement of its function, it may be removed. And if the disease has been of long standing, a cure may sometimes appear miraculous, indeed, to unprofessional persons.

There is more room for deception, in respect to the sense of hearing, than may be generally known. A deaf-mute can be taught to speak and answer questions addressed to him, without hearing a single note. Only two years since, when visiting the Institution for the Deaf and Dumb in Berlin, we had the opportunity of satisfying ourselves of this fact. By a judicious system of training, the inmates of this institution are not only taught to understand spoken language, by construing the movements of the lips of those who address then, but so to appreciate the movements of their own, by the muscular sense entirely, that they are enabled to articulate distinctly, and thus communicate with others in a satisfactory manner.

Instances may be adduced, where some of Dr. Turnbull's deaf patients actually asserted that they heard the ticking of a watch at a greater distance than persons in full possession of the sense of hearing could do. Without wishing to deceive, these persons, with their imaginations highly excited, could readily mistake the conception arising in their own minds for the sensation produced by an external agent. And those most interested in the success of the treatment, are not in a condition to detect the imposition, even were they able to apply the philosophic explanation of the phenomenon.

But it is only to the public, and not to the profession, that Dr. Turnbull makes these pretensions—implying that *he* can do what no other *mere* man can do. If the power of giving sight to the blind, hearing to the deaf, and speech to the dumb, was imparted to only ONE, who was more than man, then such power was miraculous, and cannot be arrogated by any erring mortal.

As intimated before, however, we doubt the efficacy of the means he employs, and, from our observation of the cases which have been sub-

* Kramer on Diseases of the Ear, Ed. 1838, p. 204.

jected to his treatment, we must regard him as decidedly less successful than other surgeons who make these diseases their especial study. There is already a reaction in the public mind in reference to his alleged cures. His patients are becoming weary, "with hope deferred," and many have left for their homes without having experienced the slightest amelioration of their infirmities.

